# Lithotrity in Children

**Published:** 1848-04

**Authors:** 


*Lithotrity in Children.*—In the course of some clinical remarks on a case of stone in a child four years of age, at the Hôpital des Enfans, M. Guersant, jun., referred to the following table of results of operations for stone in children by lithotomy and by lithotrity. He stated, that in fifty-one children who had been in the hospital with urinary calculi, he had performed lithotomy in thirty-seven; that in thirty of these a cure was effected, whilst only seven died; but of those seven deaths, four were attributable to intercurrent maladies, and not to the consequences of the operation. Further, that he had employed lithotrity in eight cases, of which five were cured and three died; but only one of the latter as a result of the operation. The six remaining cases of the fifty-one were examples of calculi in the urethra, readily removed.

Thus the result of both operations were equally satisfactory; but, according to the accepted practice of the hospital, lithotomy is preferred where the calculus is large, lithotrity where it is small. Lithotrity presents certain disadvantages, it is true: for instance, considerable sized fragments left in the bladder, will, on account of the ready dilatability of the neck of the bladder, and the free and strong emission of urine in children, pass into the urethra, and there get impacted, so as to render another operation necessary for their removal; however, this is not of so common an occurrence as to forbid our employment of lithotrity.

M. Guersant then gives an account of the recent case in which he operated, and where he succeeded in crushing the stone in five or six times.—*London Lancet.*

